# Virus-Infection or 5′ppp-RNA Activates Antiviral Signal through Redistribution of IPS-1 Mediated by MFN1

**DOI:** 10.1371/journal.ppat.1001012

**Published:** 2010-07-22

**Authors:** Kazuhide Onoguchi, Koji Onomoto, Shiori Takamatsu, Michihiko Jogi, Azumi Takemura, Shiho Morimoto, Ilkka Julkunen, Hideo Namiki, Mitsutoshi Yoneyama, Takashi Fujita

**Affiliations:** 1 Department of Molecular Genetics, Institute for Virus Research, Kyoto University, Kyoto, Japan; 2 Graduate School of Biostudies, Kyoto University, Yoshida-Konoe Sakyo, Kyoto, Japan; 3 Graduate School of Science and Engineering, Waseda University, Tokyo, Japan; 4 Department of Vaccination and Immune Protection, National Institute for Health and Welfare, Helsinki, Finland; 5 Division of Molecular Immunology, Medical Mycology Research Center, Chiba University, Chuo-ku, Chiba, Japan; 6 PRESTO, Japan Science and Technology Agency, Saitama, Japan; Indiana University, United States of America

## Abstract

In virus-infected cells, RIG-I-like receptor (RLR) recognizes cytoplasmic viral RNA and triggers innate immune responses including production of type I and III interferon (IFN) and the subsequent expression of IFN-inducible genes. Interferon-β promoter stimulator 1 (IPS-1, also known as MAVS, VISA and Cardif) is a downstream molecule of RLR and is expressed on the outer membrane of mitochondria. While it is known that the location of IPS-1 is essential to its function, its underlying mechanism is unknown. Our aim in this study was to delineate the function of mitochondria so as to identify more precisely its role in innate immunity. In doing so we discovered that viral infection as well as transfection with 5′ppp-RNA resulted in the redistribution of IPS-1 to form speckle-like aggregates in cells. We further found that Mitofusin 1 (MFN1), a key regulator of mitochondrial fusion and a protein associated with IPS-1 on the outer membrane of mitochondria, positively regulates RLR-mediated innate antiviral responses. Conversely, specific knockdown of MFN1 abrogates both the virus-induced redistribution of IPS-1 and IFN production. Our study suggests that mitochondria participate in the segregation of IPS-1 through their fusion processes.

## Author Summary

Virus-infections, such as influenza and chronic hepatitis C, are prominent diseases and outbreaks of newly emerging viruses are serious problems for modern society. Higher animals, including humans, are genetically equipped with mechanisms, collectively known as innate immunity, to counteract viral infections. RIG-I-like receptor (RLR), a cytoplasmic sensor, contributes to immune regulation by detecting infections by RNA viruses and triggering a series of responses which results in the activation of innate antiviral genes. Furthermore, it has been demonstrated that IPS-1, the adaptor protein of RLR, is expressed on mitochondrial outer membrane. Mitochondrion is an organelle of prokaryotic cell origin; it regulates energy production, and is involved in cell growth and cell death. Why IPS-1 is located on the mitochondrial outer membrane and how mitochondria are involved in antiviral signaling are yet to be explained clearly. In this report, we discovered that mitochondrial fusion protein MFN1 plays a novel function to mediate IPS-1 redistribution, which appears to be a critical step in RLR signaling.

## Introduction

Type I and III interferons (IFNs) play central roles in innate immune responses to viral infections [1], [2], [3], [4]. In a variety of tissues, IFN production is triggered by a cytoplasmic sensor, retinoic acid inducible gene I (RIG-I)-like Receptor (RLR), which specifically senses viral RNA and induces antiviral signaling [5], [6]. Once RLR is activated, its signal is relayed through physical interaction to IFN-β promoter stimulator 1 (IPS-1, also known as MAVS, VISA or Cardif) [7], [8], [9], [10]. IPS-1 interacts with multiple signal transducers and protein kinases that activate transcription factors to induce IFN and other cytokine genes [11]. IPS-1 is expressed on the mitochondrial outer membrane and this localization is essential for signaling to occur [9]. However the reason for this underlying mechanism is unknown. Here, we investigated the cellular distribution of IPS-1 in virus-infected cells. We observed that IPS-1 is usually distributed evenly in all mitochondria in uninfected cells, however upon viral infection or the introduction of 5′ppp-RNA, which mimics viral RNA [12], [13], [14], a redistribution of IPS-1 occurred, resulting in a speckle-like pattern on mitochondria. Furthermore, we demonstrated that a mitochondrial GTPase, Mitofusin 1 (MFN1), which regulates mitochondrial fission and fusion [15], plays a critical role in the redistribution of IPS-1, as well as in virus-induced IFN production. Our study highlights the novel mitochondrial regulatory function of specifically sorting IPS-1 and providing a signaling platform for antiviral responses.

## Results

### Dynamic redistribution of IPS-1 in virus-infected or 5′ppp-RNA-transfected cells

**Figure 1:**
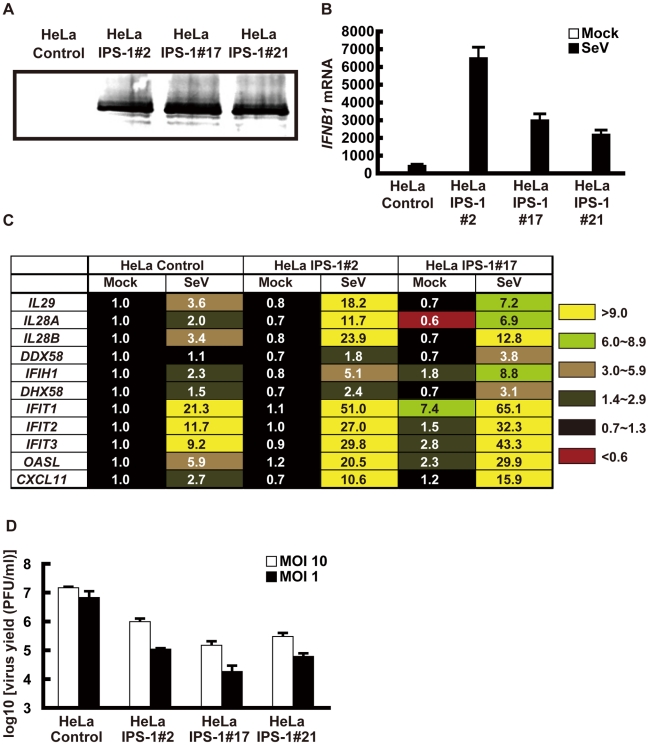
Stable HeLa cell clones expressing FLAG tagged IPS-1. **A**, Expression of FLAG-IPS-1 was examined in control and IPS-1-expressing HeLa clones (#2, 17, and 21) by immunoblotting using anti-FLAG antibody. **B**, Expression of *IFNB1* in control and IPS-1-HeLa cells was examined by quantitative real time PCR (qRT-PCR). Open and filled bars indicate mock-treated and SeV-infected cells for 12 h, respectively. Data represent means ± s.d. (n = 3). **C**, Expression profiles of cytokine and chemokine genes in control and IPS-1-HeLa cells. Total RNA extracted from indicated cells mock-treated or SeV-infected for 12 h was subjected to analysis using a DNA microarray. Relative mRNA levels using a control expression of 1.0 are shown. **D**, Replication of EMCV in control and IPS-1-HeLa clones. The indicated cell clones were infected with EMCV at a MOI of 1 or 10. The viral titer in the culture medium at 24 h post-infection was determined with the plaque assay. Data represent means ± s.d. (n = 3).

To examine the localization of IPS-1 during viral infections, we generated HeLa cell lines stably expressing FLAG-tagged IPS-1 (IPS-1-HeLa clones, Fig. 1). Although the temporary expression of wild type (wt) IPS-1 results in constitutive signaling [7], [8], [9], [10], the stable cell lines did not exhibit the constitutive activation of downstream target genes. However, upon infection with the Sendai virus (SeV), the cells exhibited increased expression of IFN and chemokine genes (*IFNB1*, *IL29*, *IL28A*, *IL28B* and *CXCL11*) and interferon-stimulated genes (*DDX58*, *IFIH1*, *DHX58*, *IFIT1-3*, and *OASL*) (Fig. 1B and C). Furthermore, the IPS-1-HeLa clones exhibited diminished susceptibility to Encephalomyocarditis virus (EMCV) replication (1 to 2 log) (Fig. 1D). The low basal activity and elevated signaling after SeV-infection suggest that FLAG-IPS-1 is under a regulatory control similar to that of endogenous IPS-1.

**Figure 2:**
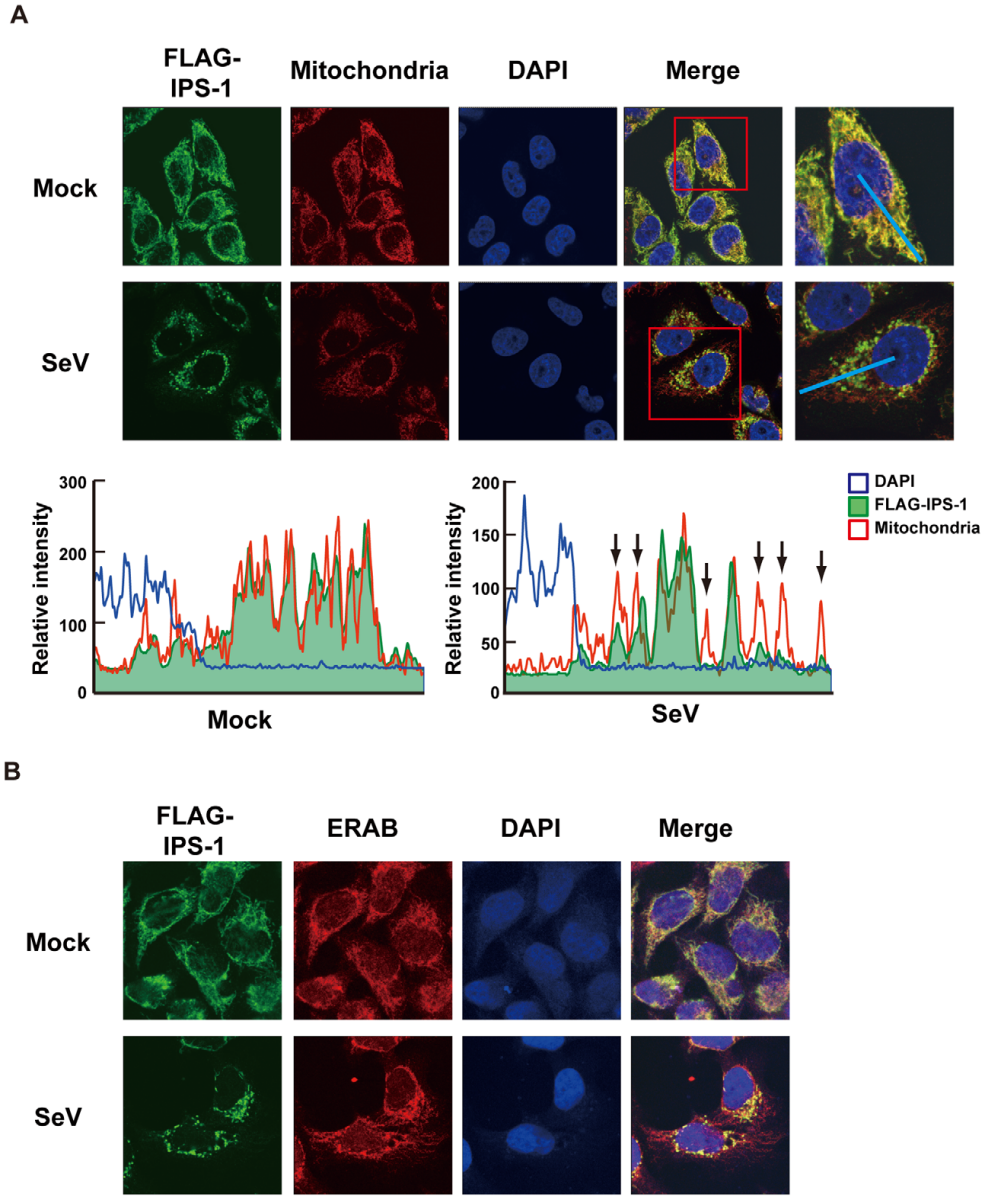
Redistribution of IPS-1 in SeV-infected cells. **A**, The IPS-1-HeLa clone #2 was mock-treated or infected with SeV for 12 h and stained with MitoTracker (Mitochondria) and anti-FLAG antibody (FLAG-IPS-1). Nuclei were visualized by staining with DAPI throughout this study. The fluorescent image was quantified in the area indicated by blue line (right most panel). Quantification results from mock- or SeV-infected cells are shown at the bottom. Fluorescence of DAPI corresponds to area in the nucleus. The mitochondria heavily stained with MitoTracker but lightly stained with anti-FLAG are shown by arrows. **B**, IPS-1-HeLa cells were mock-treated or infected with SeV for 12 h. Cells were stained with anti-FLAG antibody (FLAG-IPS-1) and anti-ERAB antibody (ERAB).

**Figure 3:**
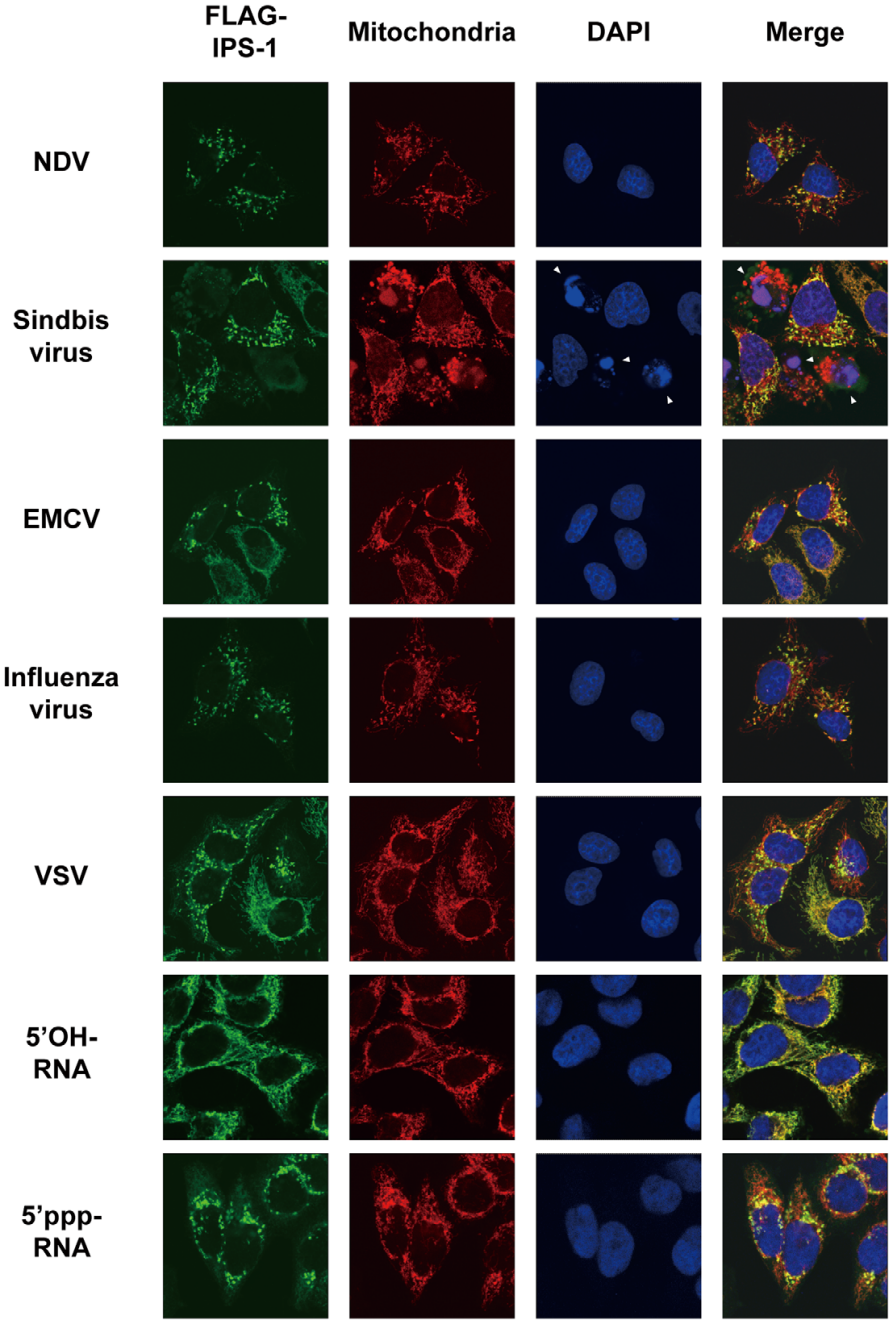
Redistribution of IPS-1 induced by virus-infection and 5′ppp-RNA-transfection. IPS-1-HeLa cells were infected with the indicated viruses or transfected with 5′OH-RNA or 5′ppp-RNA chemically synthesized by in vitro transcription using T7 RNA polymerase. At 12 h post-infection or -transfection, the cells were stained with anti-FLAG antibody and MitoTracker (Mitochondria). Arrowheads show dead cells with shrunk nuclei in Sindbis virus-infected cells.

Like endogenous IPS-1, FLAG-tagged IPS-1 is expressed on mitochondria in uninfected cells as shown by co-staining with MitoTracker (Fig. 2A, Mock). However, compared to the even cytoplasmic staining in the mock-infected cells, the staining pattern of IPS-1 became noticeably speckled in SeV-infected cells (Fig. 2A, SeV). Quantification of the fluorescence image revealed that mitochondria heavily stained with MitoTracker but lightly stained with anti-FLAG antibody were produced in SeV-infected cells. This redistribution was also observed with another mitochondrial marker, endoplasmic reticulum-associated amyloid β-peptide-binding protein (ERAB) (Fig. 2B), and different viruses (Newcastle disease virus (NDV), Sindbis virus, EMCV, Influenza virus, and Vesicular stomatitis virus (VSV)) (Fig. 3).

**Figure 4:**
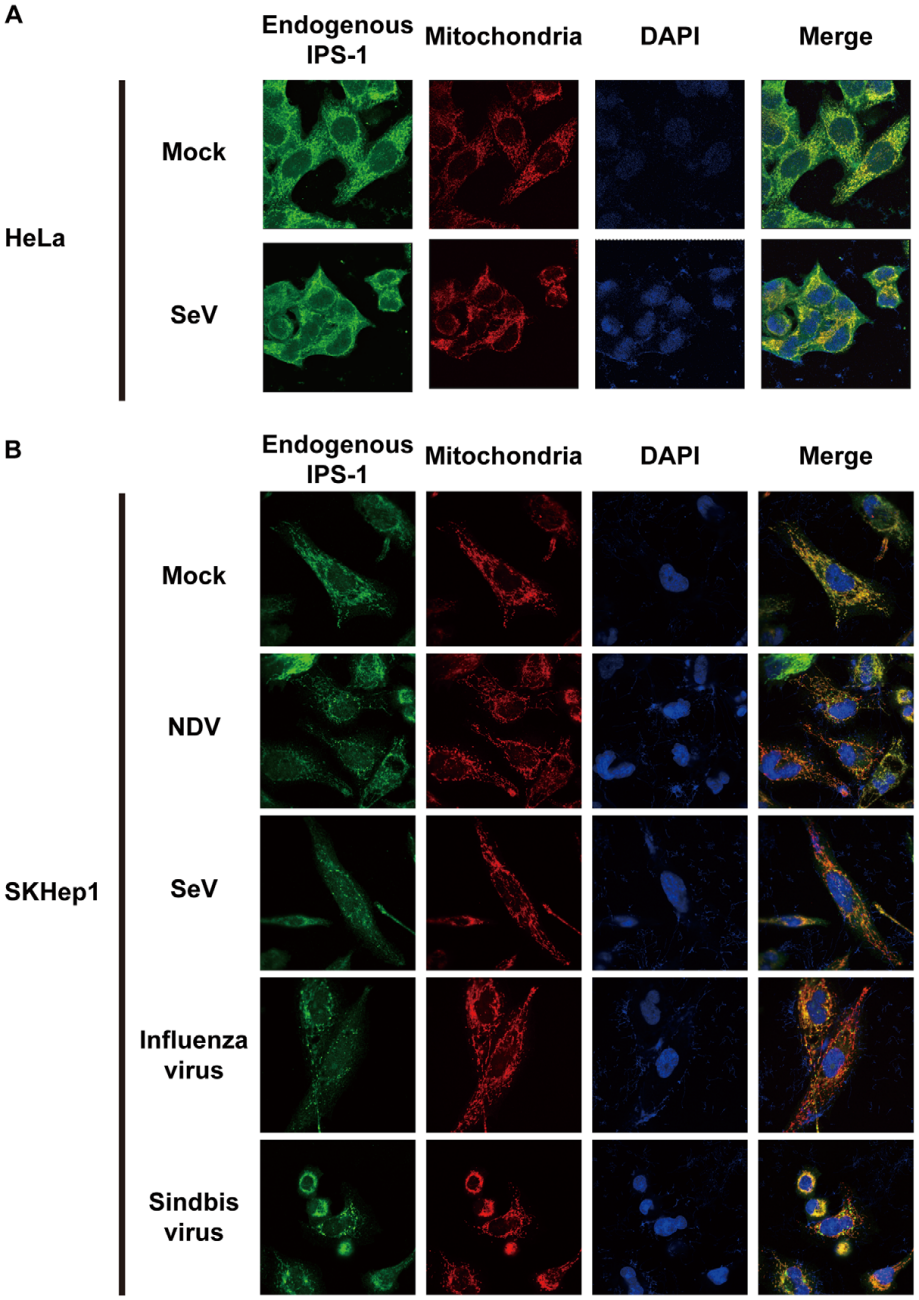
Redistribution of endogenous IPS-1 in virus-infected cells. **A**, HeLa cells were infected with Mock or SeV for 12 h. The cells were stained with anti-IPS-1 antibody and MitoTracker (Mitochondria). **B**, SKHep1 cells were infected with NDV, SeV, Influenza virus, or Sindbis virus for 12 h. The cells were stained with anti-IPS-1 antibody and MitoTracker (Mitochondria).

We also examined the distribution of IPS-1 in 5′ppp-RNA-transfected cells. Unlike synthetic single stranded RNA (5′OH-RNA), 5′ppp-RNA is a chemical ligand for RIG-I and is known to mimic viral signaling [12], [13], [14]. Interestingly, as with a viral infection, 5′ppp-RNA induced a redistribution of IPS-1, suggesting that the redistribution was triggered through RIG-I signaling. It is worth noting that EMCV, which selectively activates another RLR, melanoma differentiation-associated gene 5 (MDA5), also caused the redistribution of IPS-1, suggesting that this effect is common to RLRs. We suspected that IPS-1-HeLa cells exhibit enhanced redistribution of IPS-1 due to enhanced signaling (>10 IFN-β mRNA accumulation, Fig. 1B). This led us to analyze the distribution pattern of endogenous IPS-1 in HeLa cells, and we observed that the distribution pattern of endogenous IPS-1 changed in SeV-infected cells, although exclusive staining by mitochondrial marker was not observed (Fig. 4A). Similar to IPS-1-HeLa cells, we observed that hepatocellular carcinoma SKHep1 cells NDV, SeV, Influenza virus, or Sindbis virus infection also induced a speckled staining pattern in endogenous IPS-1 (Fig. 4B), and displayed enhanced IRF-3 dimerization when compared with HeLa cells (our unpublished data). This suggests that the redistribution is not simply an artifact due to the overexpression of FLAG-IPS-1.

### Localization of viral nucleocapsid, RIG-I, and IPS-1

**Figure 5:**
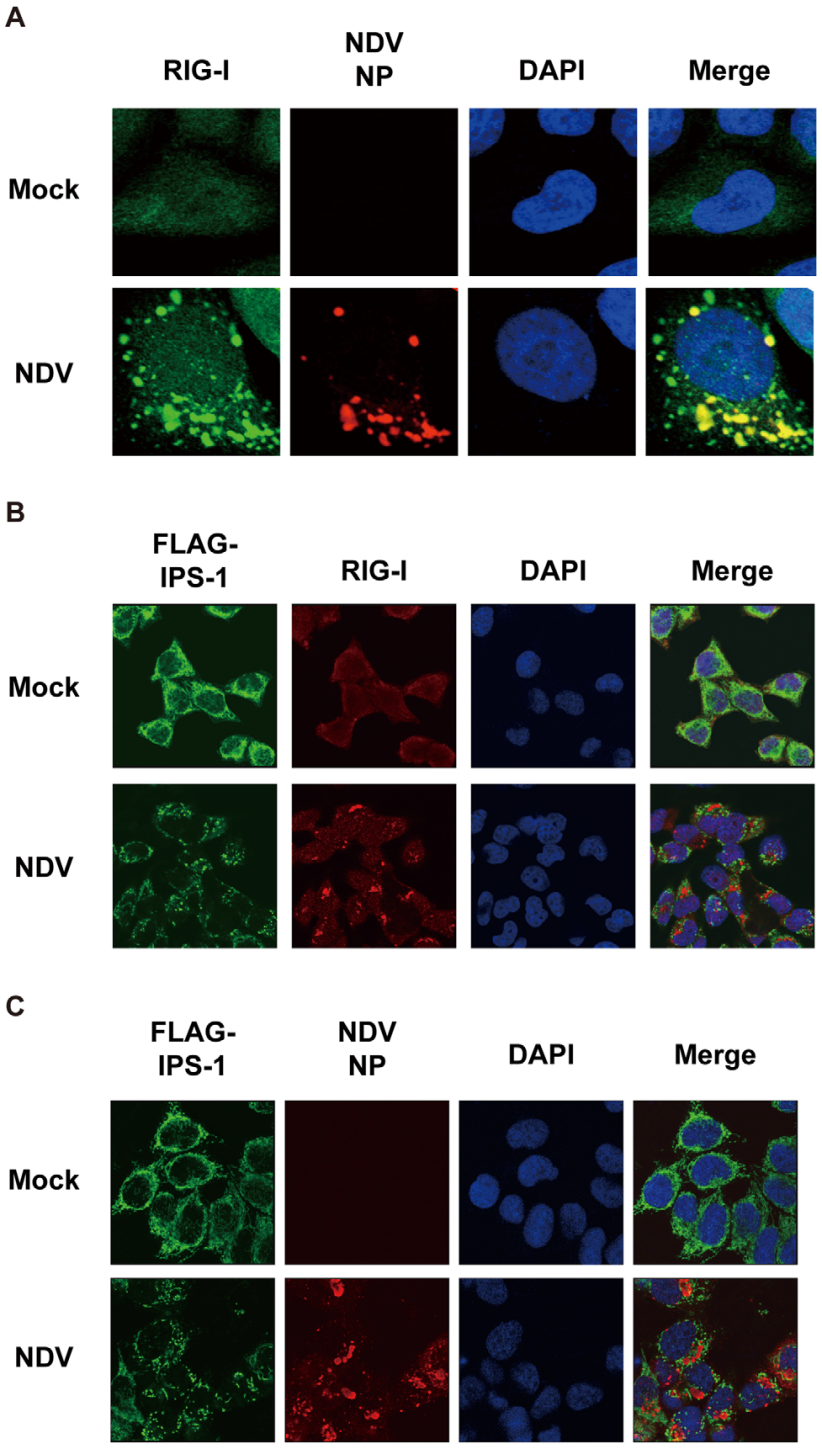
Localization of viral nucleocapsid, RIG-I, and IPS-1. **A**, HeLa cells were infected with NDV for 12 h and stained with anti-RIG-I antibody (RIG-I) and anti-NP antibody (NDV NP). **B** and **C**, IPS-1-HeLa cells were infected with NDV for 12 h and stained with anti-FLAG antibody and anti-RIG-I antibody or anti-NP antibody.

**Figure 6:**
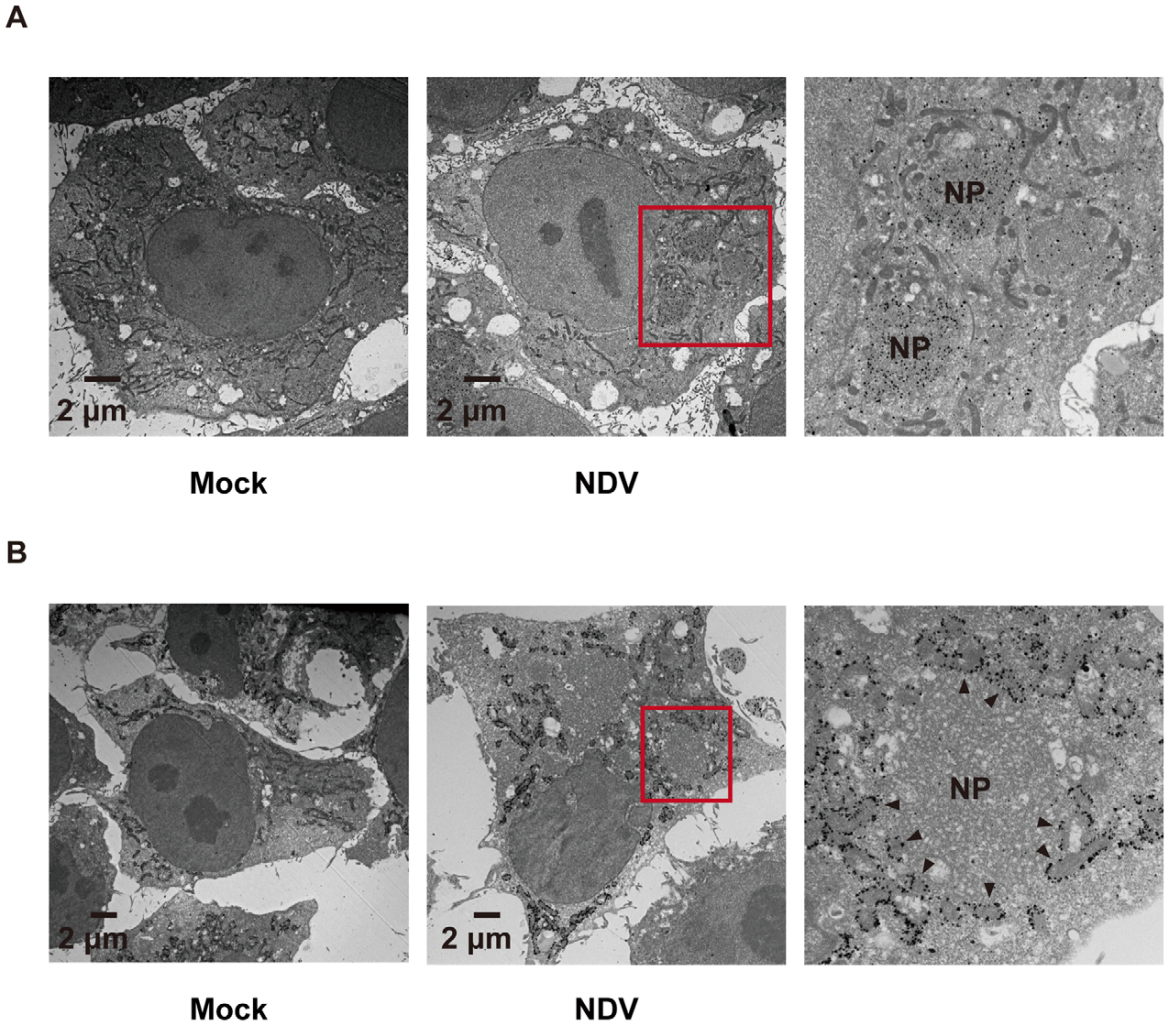
Localization of IPS-1 and mitochondria. **A**, IPS-1-HeLa cells infected with NDV for 9 h were fixed, stained with anti-NP antibody, and subjected to ultrathin sectioning as shown in the Methods. The area enclosed by a red rectangle is enlarged. NP: NP foci stained with the anti-NP antibody were visualized using gold particles. **B**, IPS-1-HeLa cells infected with NDV for 9 h were fixed, stained with anti-FLAG antibody, and subjected to ultra thin sectioning. The area enclosed by a red rectangle is enlarged. NP: morphologically similar structures are in **A**. IPS-1 was visualized using gold particles. The arrowheads indicate boundaries between IPS-1 and NP foci.

In order to activate RLR signaling, we used NDV to infect cells because it is available an anti-nucleocapsid protein (NP) antibody, a probe for the viral RNA-NP complex. NDV infection resulted in foci of NP in the cytoplasm and induced foci of RIG-I to form (Fig. 5A) [16]. RIG-I was evenly distributed in the cytoplasm, however some of the foci co-localized with those of NP (Fig. 5A). A similar formation of foci and co-localization with viral nucleoprotein complex was observed with other viruses (Ko.O. unpublished observations). IPS-1 accumulated on the periphery of the foci of RIG-I (Fig. 5B) and NP (Fig. 5C). We speculate that activated RIG-I recruits IPS-1, because RIG-I and IPS-1 interacted with each other through CARD-CARD interaction [7], [8]. IPS-1 did not co-localize with RIG-I nor NP presumably because mitochondria do not penetrate these foci nor is IPS-1 released from mitochondria. Immunoelectron microscopy using the anti-NP antibody clearly identified the NP foci (Fig. 6A), and anti-FLAG staining (Fig. 6B) showed that mitochondrial IPS-1 accumulated on the periphery of NP foci in NDV infected cells.

### A dominant negative mutant of RIG-I does not induce IPS-1 redistribution

**Figure 7:**
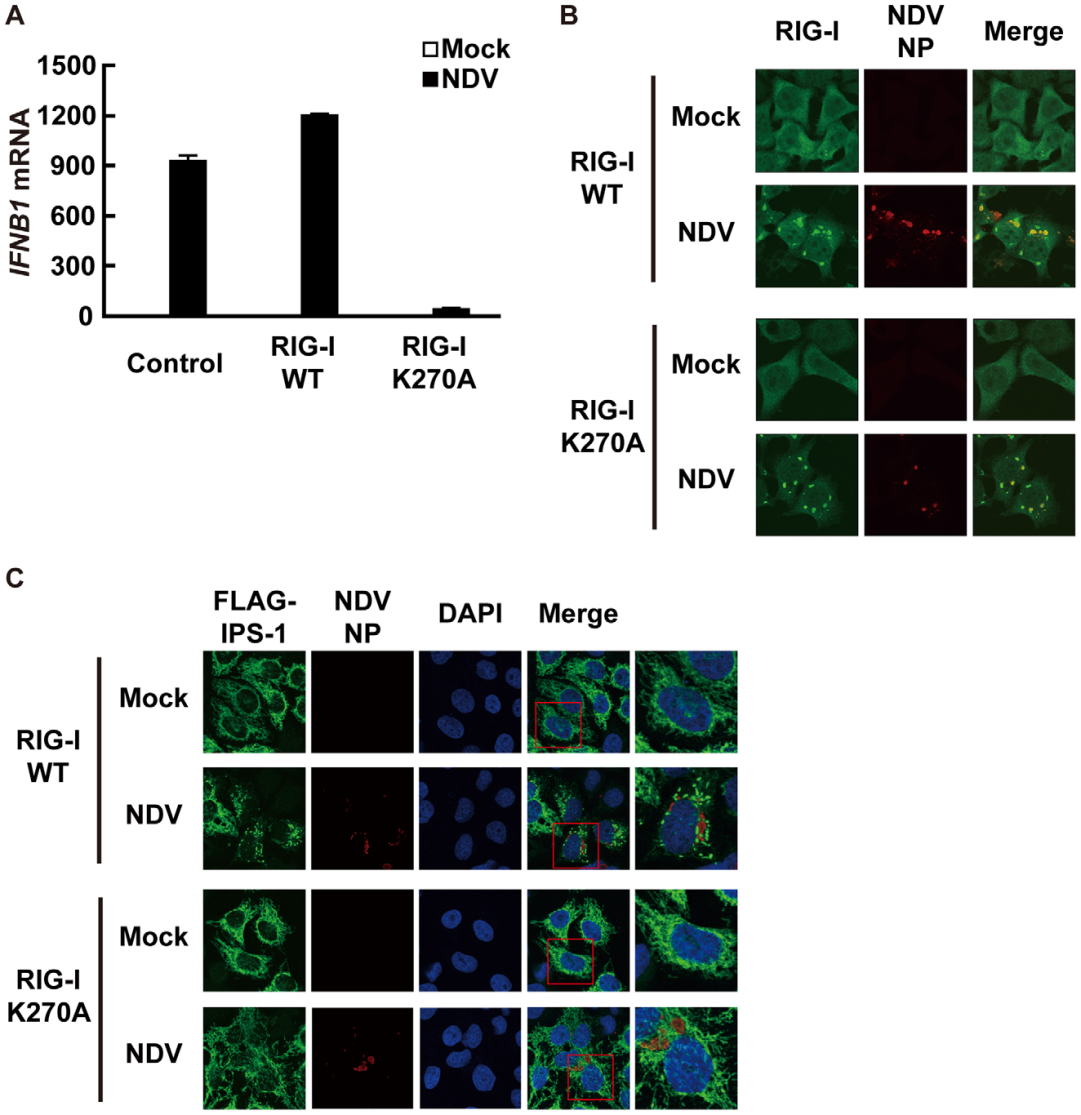
A dominant negative mutant of RIG-I fails to induce IPS-1 redistribution. **A**, IPS-1-HeLa cells stably expressing wild-type human RIG-I (RIG-I WT) or mutant RIG-I (RIG-I K270A) were mock-treated or infected with NDV for 12 h and expression of *IFNB1* mRNA was analyzed by qRT-PCR. Open and filled bars indicate RNA samples from mock-treated and NDV-infected cells, respectively. Data represent means ± s.d. (n = 3). **B**, IPS-1-HeLa cells expressing RIG-I WT or RIG-I K270A were infected with NDV for 12 h and stained with anti-RIG-I antibody and anti-NP antibody. RIG-I staining is diffuse in uninfected cells however infection by NDV produced RIG-I foci. Some RIG-I foci are co-localized with NDV NP foci. **C**, IPS-1-HeLa cells expressing RIG-I WT or RIG-I K270A were infected with NDV for 12 h and IPS-1 redistribution was examined. IPS-1 and NP were stained with anti-IPS-1 antibody and anti-NP antibody, respectively. The area enclosed by the red rectangle is enlarged at the right. Although the redistributed IPS-1 surrounds NP foci in RIG-I WT cells, K270A mutation of RIG-I failed to induce the redistribution of IPS-1, but not the formation of NP foci.

To determine if the observed redistribution of IPS-1 is functionally relevant, we used a point mutant of RIG-I (K270A), which normally recognizes ligand RNA but functions as a dominant negative inhibitor (Fig. 7A) [14]. It was observed that NDV infection induced foci of both wt and K270A RIG-I to form (Fig. 7B), however wt but not K270A promoted the speckled staining pattern of IPS-1 (Fig. 7C). The results indicate that the redistribution of IPS-1 is strongly correlated with the activation of antiviral signaling.

### Mitofusin 1, but not Mitofusin 2, plays a critical role in RIG-I-induced antiviral signaling

**Figure 8:**
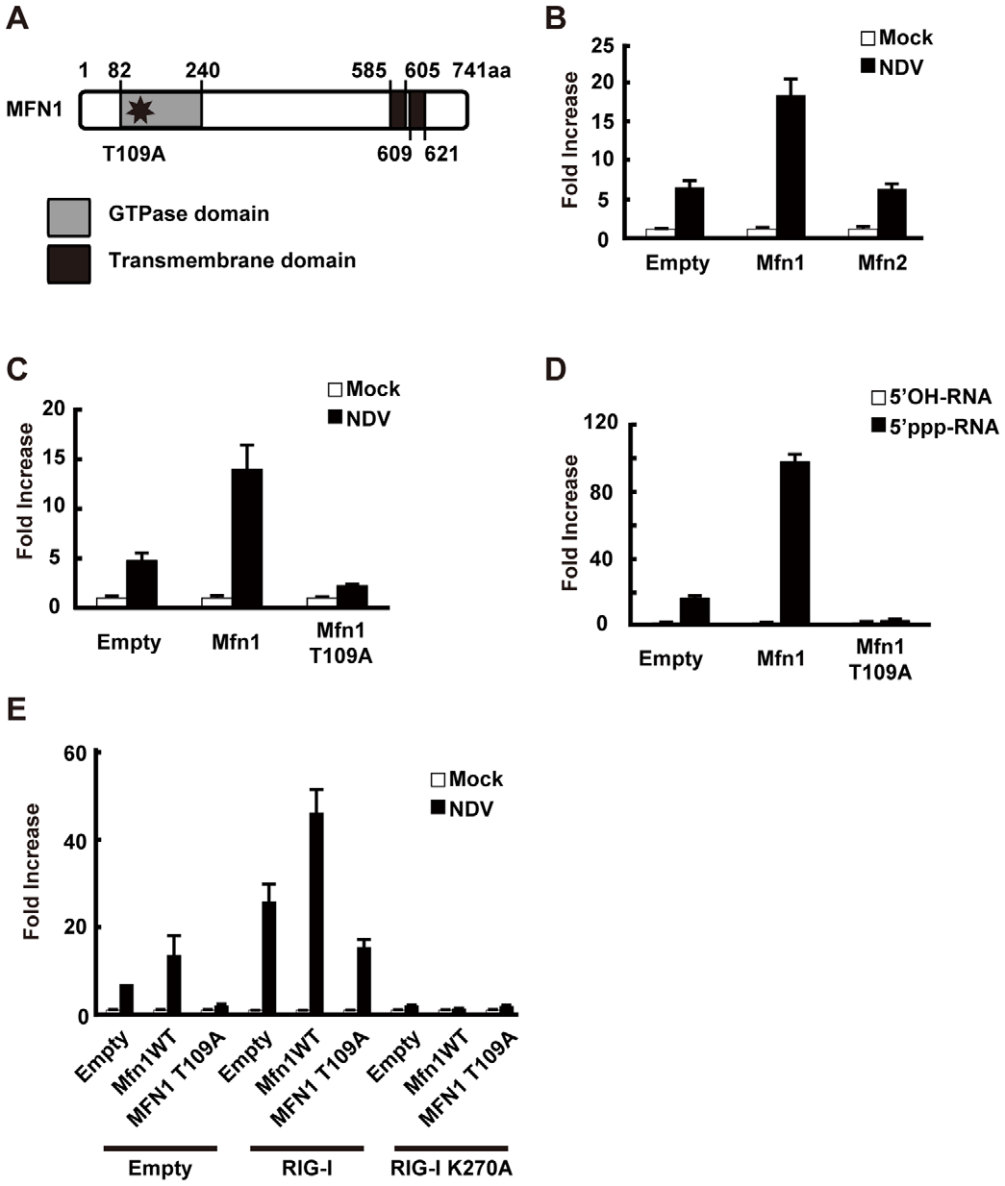
MFN1 is involved in antiviral signaling. **A**, Schematic representation of the MFN1 domain. **B**, L929 cells were transfected with a virus-responsive reporter gene (p-125 Luc) and either an empty vector (Empty), an expression vector for MFN1, or an expression vector for MFN2 as indicated. 48 h after the transfection, cells were mock-treated or infected with NDV. Luciferase activity was determined at 12 h after infection. **C** and **D**, L929 cells were transfected with a virus-responsive reporter gene (p-125 Luc) and either an empty vector (Empty) or an expression vector for MFN1 or its mutant (MFN1 T109A) as indicated. At 48 h after transfection, cells were mock-treated, infected with NDV, or transfected with 5′OH-RNA or 5′ppp-RNA. Luciferase activity was determined at 12 h (**C**) or 9 h (**D**) after induction. **E**, L929 cells were transfected with a virus-responsive reporter gene (p-125 Luc) and combinations of the indicated vectors. 48 h after the transfection, cells were mock-treated or infected with NDV. Luciferase activity was determined 12 h after infection.

RIG-I was originally identified by screening an expression cDNA library [17]. In addition to the cDNA encoding RIG-I, there were several other candidate cDNA clones which enhance virus-responsive reporter activity. Two of the independent clones encoded a full-length protein, Mitofusin 1 (MFN1). Human MFN1 is composed of 741 amino acids and domains of GTPase and transmembranes (Fig. 8A). MFN1 together with its related protein Mitofusin 2 (MFN2) is expressed on the outer membrane of mitochondria and regulates mitochondrial dynamics [18], [19]. Hyper- and hypo-functioning of either MFN1 or MFN2 result in elongated/aggregated and fragmented mitochondria, respectively. GTPase activity was previously shown to be essential for mitochondrial morphological change, particularly the fragmentation of mitochondria induced by a GTP-binding-deficient mutant of MFN1 (MFN1 T109A) [18].

**Figure 9:**
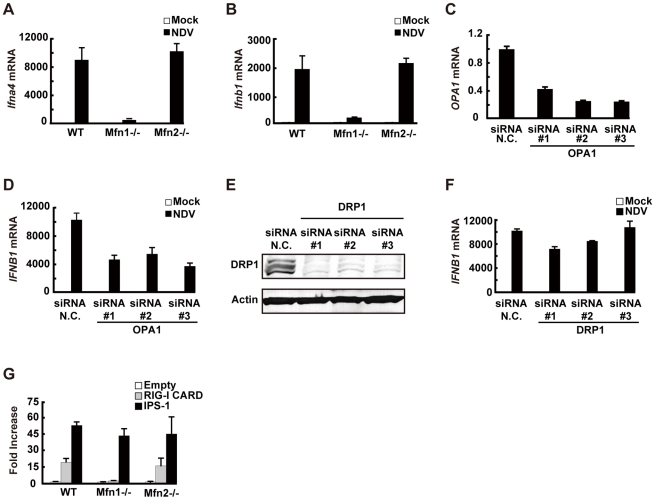
MFN1 plays a critical role in RIG-I–induced signaling. **A** and **B**, Wild-type (WT) MEFs and Mfn1 or Mfn2-knockout MEFs were infected with NDV for 9 h. The levels of endogenous *Ifna4* (**A**) and *Ifnb1* (**B**) mRNA were quantified by qRT-PCR. **C**, HeLa cells were transfected with negative control (N.C.) or hOPA1-targeted siRNA (#1–#3) for 72 h, and the expression of *OPA1* mRNA was analyzed by qRT-PCR. **D**, HeLa cells were transfected with N.C. siRNA or hOPA1-targeted siRNA. 72 h after transfection, cells were infected with NDV for 12 h. *IFNB1* mRNA expression was quantified by qRT-PCR. **E**, HeLa cells were transfected with N.C. siRNA or hDRP1-targeted siRNA (#1–#3) for 72 h, and the knockdown of endogenous DRP1 was analyzed by Western blotting using anti-DRP1 antibody. **F**, HeLa cells were transfected with N.C. siRNA or hDRP1-targeted siRNA. At 72 h after transfection, cells were infected with NDV for 12 h. *IFNB1* mRNA expression was quantified by qRT-PCR. **G**, WT and Mfn1 or Mfn2-knockout MEFs were transfected with a virus-responsive reporter gene (p-125 Luc) with either an empty vector (Empty) or an expression vector for RIG-I CARD or IPS-1. Luciferase activity was determined 48 h after transfection. Data represent means ± s.d. (n = 3).

Consistent with the screening results, overexpression of MFN1, but not MFN2, augmented IFN-β promoter activity (Fig. 8B). The GTPase activity is involved in this MFN1 function, since MFN1 T109A significantly inhibited the signaling induced by NDV or 5′ppp-RNA (Fig. 8C and D). It is worth noting that overexpression of MFN1, which results in elongated mitochondria, is not by itself sufficient to deliver the signal. To confirm that the increased signaling observed by MFN1 overexpression was correlated with RIG-I activation, we transfected cells with a combination of RIG-I and MFN1. The RIG-I/MFN1 combination showed enhanced IFN-β promoter activity, but the RIG-I K270A mutant/MFN1 combination failed to do so (Fig. 8E). MEFs derived from mice with disrupted *Mfn1* or *Mfn2* gene was used to confirm the specific involvement of MFN1 in virus-induced antiviral signaling (Fig. 9A and B). The results indicated that MFN1, but not MFN2, is essential for the signal transduction mediated by RIG-I.

We examined other regulatory proteins for mitochondrial fission/fusion mechanism. Optic atrophy protein 1 (OPA1) is expressed on, and implicated in the fusion of the mitochondrial inner membrane [20]. Three independent siRNA targeting OPA1, down-regulated OPA1 expression (Fig. 9C) and partially (up to 50%) blocked NDV-induced signaling (Fig. 9D). However, the knockdown of dynamin-related protein 1 (DRP1) (Fig. 9E), which regulates mitochondrial fission [21] resulting in elongated mitochondria, did not have a significant effect (Fig. 9F). To explore the site where MFN1 is active, we temporarily overexpressed the dominant active RIG-I (RIG-I CARD) [17] or IPS-1 in wt and Mfn-knockout MEFs. Unlike the signal generated by the overexpression of IPS-1, the signal generated by overexpression of the RIG-I tandem caspase recruitment domain (CARD) clearly required MFN1. MFN1 however, is dispensable if IPS-1 is overexpressed (Fig. 9G). Again, MFN2 exhibited little influence on the signaling triggered by either stimulus. These results indicate that MFN1, but not MFN2, is essential for signal transduction mediated by RIG-I and IPS-1.

### Physical interaction between IPS-1 and MFN1

**Figure 10:**
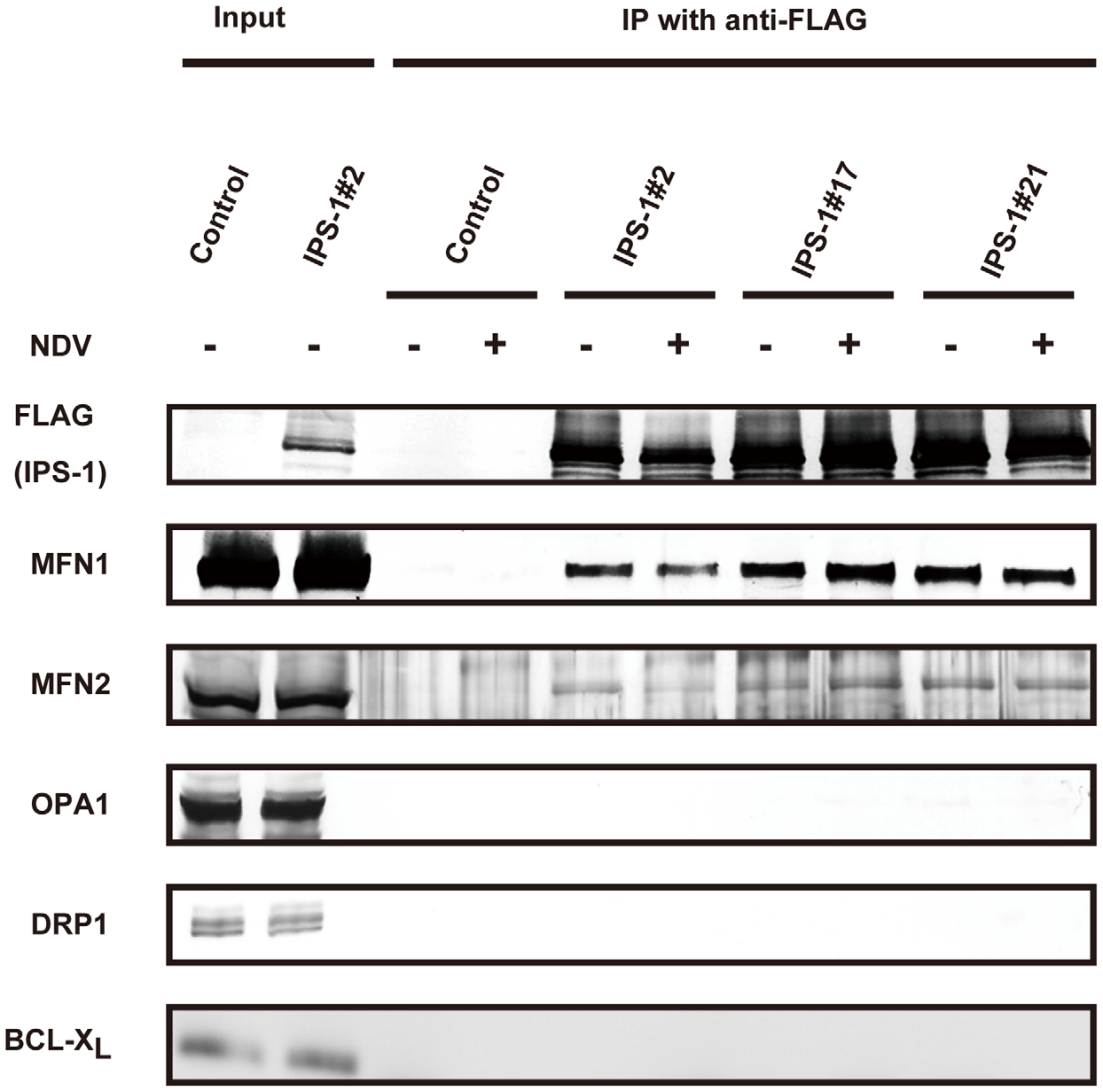
IPS-1 interacts with MFN1 and MFN2. IPS-1-HeLa cells were infected with NDV for 12 h, and then FLAG-IPS-1 was immunoprecipitated with anti-FLAG antibody. Co-immunoprecipitated MFN1 and MFN2 were detected by anti-MFN1 antibody and anti-MFN2 antibody, respectively. Neither OPA1 nor DRP1 was co-immunoprecipitated with FLAG-IPS-1. Mitochondrial protein BCL-Xl was used as a control and was also examined by anti-BCLXl antibody.

To explore the molecular mechanism of how IPS-1 is regulated by MFN1, co-immunoprecipitation was performed using cells stably expressing IPS-1. FLAG-IPS-1 was precipitated by anti-FLAG and the associated proteins were analyzed by immunoblotting (Fig. 10). Both MFN1 and MFN2 constitutively associated with IPS-1 in the cells, but an unrelated mitochondrial outer membrane protein, BCL-XL, did not associate with IPS-1. Furthermore, OPA1 and DRP1 did not co-immunoprecipitate with FLAG-IPS-1. These data suggest that IPS-1 selectively associates with MFN1 and MFN2.

### Knockdown of MFN1 inhibits the redistribution of IPS-1 induced by viral infection

**Figure 11:**
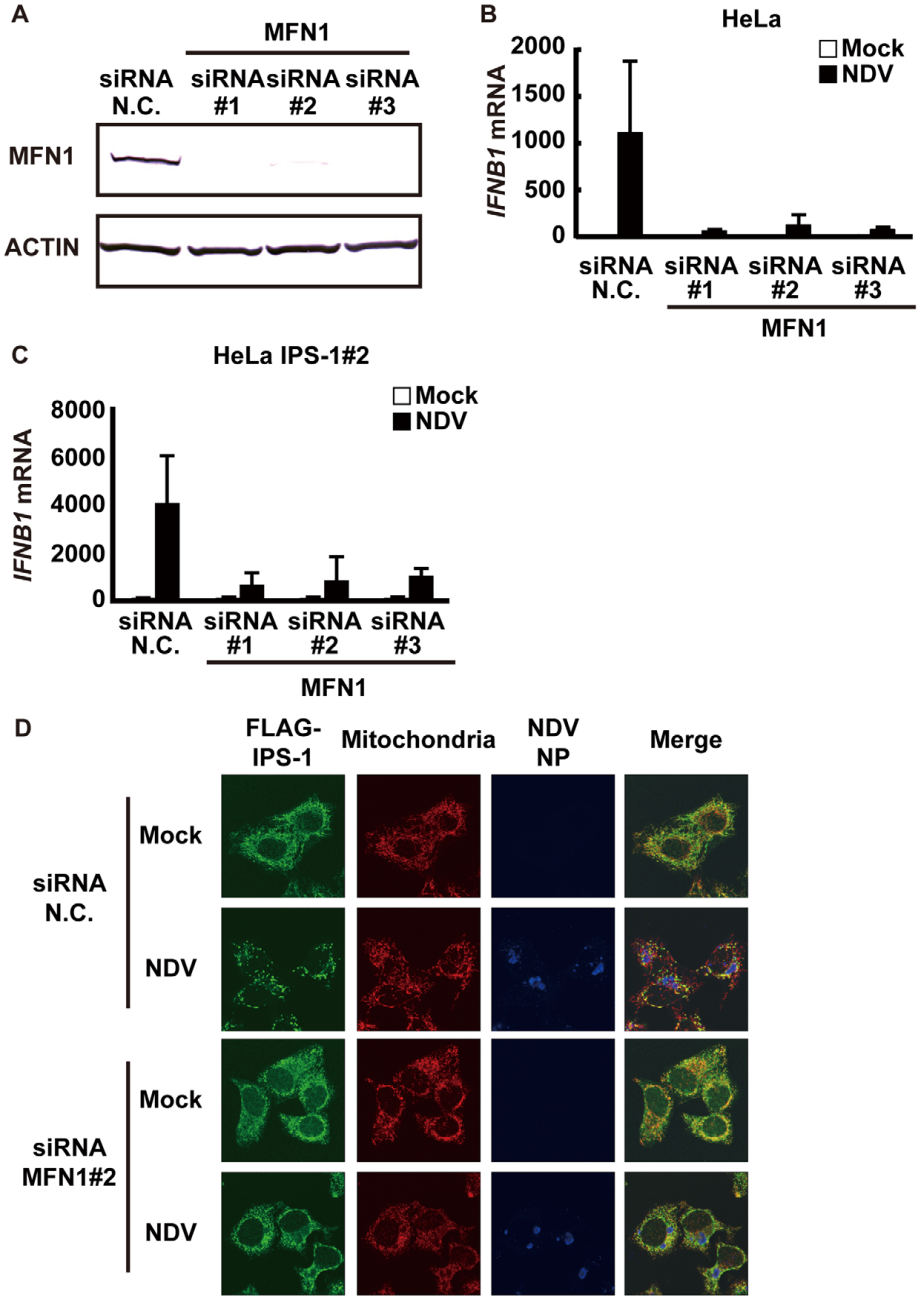
Knockdown of MFN1 inhibits the redistribution of IPS-1 induced by NDV infection. **A**, HeLa cells were transfected with negative control (N.C.) or hMFN1-targeted siRNA (#1–#3) for 48 h, and the knockdown of endogenous MFN1 was analyzed by Western blotting using anti-MFN1 antibody. **B**, Cells transfected with siRNA as shown in **a** were infected with NDV for 12 h, and endogenous *IFNB1* mRNA expression was quantified by qRT-PCR. Data represent means ± s.d. (n = 3). **C**, IPS-1-HeLa cells transfected with N.C. or hMFN1-targeted siRNA were infected with NDV for 12 h, and endogenous *IFNB1* mRNA expression was quantified by qRT-PCR. Data represent means ± s.d. (n = 3). **D**, IPS-1-HeLa cells transfected with N.C. or hMFN1-targeted siRNA#2 for 48 h. Cells were infected with NDV for 12 h and stained with anti-FLAG antibody (FLAG-IPS-1), anti-NP antibody (NDV NP), and MitoTracker (Mitochondria).

**Figure 12:**
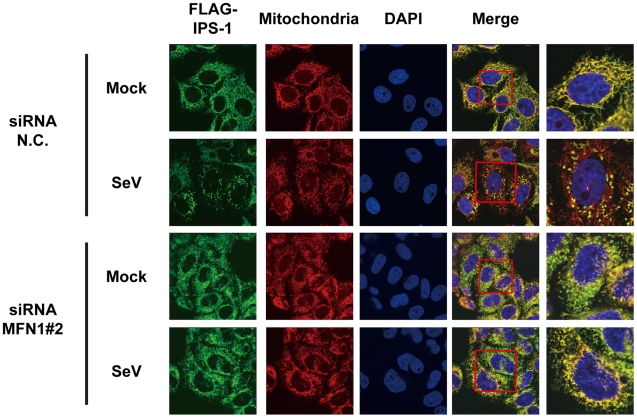
Knockdown of MFN1 inhibits the redistribution of IPS-1 induced by SeV infection. IPS-1-HeLa cells transfected with negative control (N.C.) or hMFN1-targeted siRNA#2 for 48 h. Cells were infected with SeV for 12 h and stained with anti-FLAG antibody (FLAG-IPS-1), MitoTracker (Mitochondria), and DAPI. The area enclosed by the red rectangle is enlarged at the right.

Next, we examined what effect the knockdown of MFN1 would have on the virus-induced redistribution of IPS-1. Three independent siRNA efficiently knocked down MFN1 expression (Fig. 11A) resulting in a strong inhibition of the NDV-induced IFN-β gene expression in HeLa cells and IPS-1-HeLa cells (Fig. 11B and 11C). This once again suggests that IPS-1-HeLa cells tend to behave like normal cells. Upon NDV infection, IPS-I displayed a speckled staining pattern in control cells, but not in the MFN1-knockdown cells (Fig. 11D). Though the intensity of NP staining did not increase, MFN1-knockdown significantly inhibited IFN gene activation. This correlates with prior observations that although IFN production is inhibited by LGP2 overexpression, viral yield does not increase [22]. When control siRNA-treated cells were infected with NDV, a redistribution of IPS-1 was observed (69.3±15.7% of cells positive for NP). In MFN1-knockdown cells, although NDV infection resulted in the formation of NP foci, IPS-1 redistribution did not occur (4.5±1.3% of cells positive for NP). A similar effect was observed when MFN1-knockdown cells were infected with SeV (Fig. 12). These results strongly suggest that MFN1 is critical to the redistribution of IPS-1 triggered by RIG-I mediated sensing of viral RNA.

## Discussion

RIG-I mediated antiviral signaling is a critical antiviral response which is initiated when the RIG-I sensor recognizes viral RNA. A signal is relayed to IPS-1, a mitochondrial regulator which delivers the signal downstream. Interestingly, the IPS-1-HeLa clones in this study exhibited very low basal expression of IFN genes, which led us to speculate that IPS-I inhibitory protein(s) is up regulated in these clones. We also examined the expression level of NLRX1, an IPS-1 inhibitor [23], and noted no change in its expression level (not shown). Similarly, levels of MFN1 and MFN2 did not change in the IPS-1-HeLa clones. (Fig. 10, input).

We observed that the IPS-1 level did not change for up to 12 h in virus-infected cells and no specific modification of IPS-1 was identified up to that point. We therefore hypothesize that the activation status of IPS-1 is determined by its localization pattern. We speculate that the mechanism of mitochondrial fusion is mediated by MFN1, and that IPS-1 translocates from some mitochondria and finally accumulates densely on others. On the other hand, forced overexpression of full-length IPS-1 results in constitutive signaling [7], [8], [9], [10]. There is no clear explanation why non-physiological overexpression can by-pass the virus-induced signaling, however, it can be concluded that transient IPS-1 overexpression may quantitatively override the hypothetical inhibitor for IPS-1 (above). Under these conditions, MFN1 is dispensable (Fig. 9G). Consistent with this, artificial aggregation of IPS-1 by cross-linking induced the signaling to activate IFN genes (Tang, E. D. and Wang, C. D. [24] and our unpublished observation). We speculate that under physiological conditions, viral infection induces the local accumulation of IPS-1 (corresponds to “active IPS-1”) on mitochondria. These mitochondria with locally accumulated IPS-1 may function as a platform to recruit downstream molecules.

MFN1 and MFN2 are structurally similar and both occur on the outer membrane of mitochondria. Their functions however are not redundant, as the single knockout of either produces a certain mitochondrial phenotype [19]. Recently two papers were published concerning MFN function in RIG-I-mediated antiviral responses. Yasukawa et al. reported that MFN2 strongly interacts with IPS-1 thereby blocking its function, however MFN1 does not interact with IPS-1 and exhibits no effect [25]. These observations are clearly inconsistent with ours. The report by Castanier et al. however, is consistent with our finding that MFN1, but not MFN2, positively regulates IPS-1 [26]. Most importantly, our knockout results are clearly consistent with their knockdown results (Fig. 9A and B). Castanier et al. observed that a particular variant of SeV (H4) causes elongation of mitochondria, however they did not demonstrate whether this morphological change is common to other viral infections. Unlike Castanier et al. we did not observe mitochondrial elongation by viral infections nor 5′-pppRNA transfection (Fig. 3). The knockdown of a mitochondrial inner membrane protein OPA1 blocked virus-induced signaling (Fig. 9D); thereby indicating that mitochondrial fusion may be necessary for the activation of IPS-1. Castanier et al. observed that knockdown of DRP1 or FIS1 causes mitochondrial elongation and IPS-1's association with STING, an antiviral signaling adaptor. However, neither the mitochondrial elongation nor the IPS-1-STING interaction is sufficient to activate the signaling.

**Figure 13:**
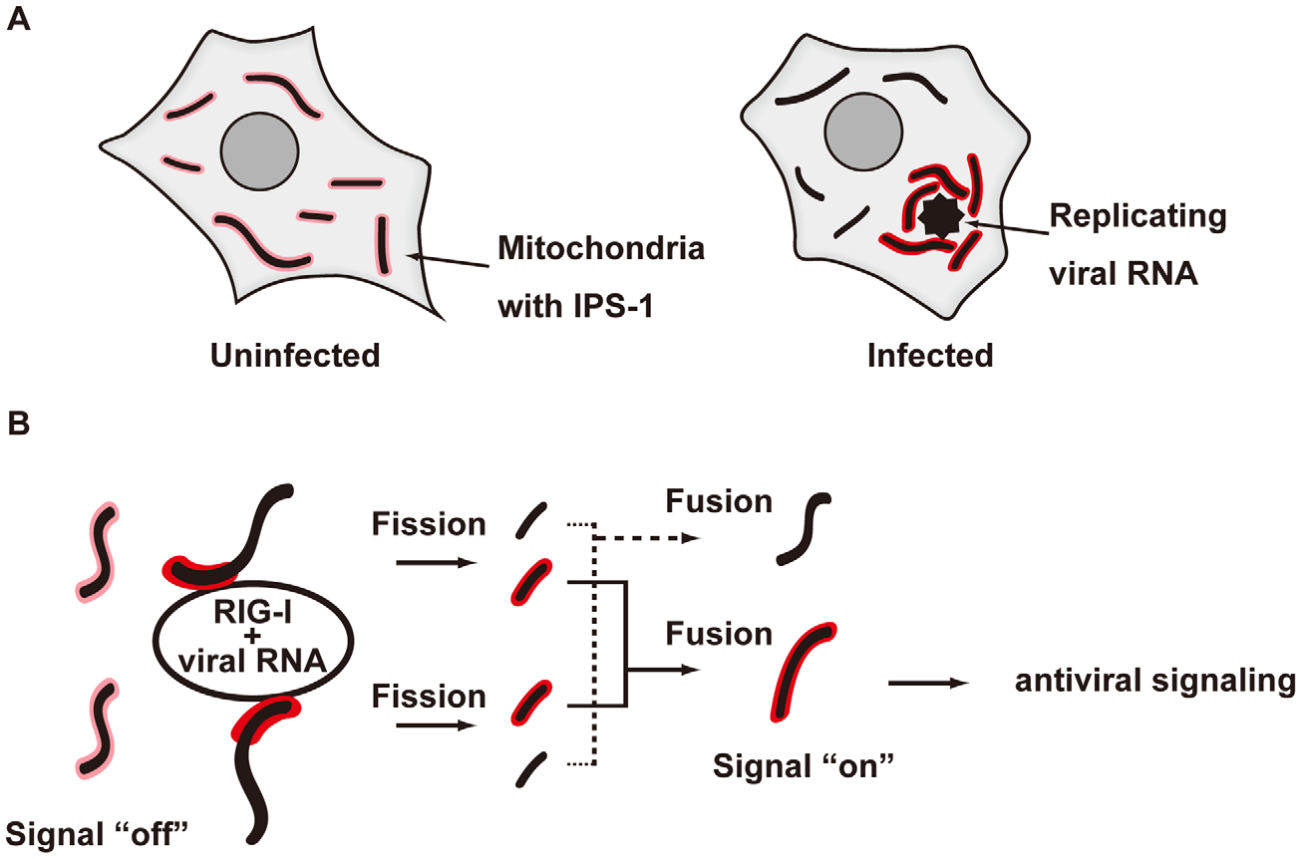
Model for the redistribution of IPS-1 mediated by mitochondrial organization. **A**, Schematic representation of the redistribution of IPS-1 induced by viral infection. In uninfected cells, IPS-1 is evenly distributed in all mitochondria (left). In infected cells, foci of viral nucleoprotein form, which are surrounded by redistributed IPS-1 and mitochondria (right). **B**, A model for the redistribution of IPS-1 mediated by mitochondrial organization. Initially, IPS-1 is distributed evenly in mitochondria (left). Viruses replicate in restricted compartments in the cells, viral RNA accumulates, and then RIG-I re-localizes to these compartments. Viral RNA induces a conformational change of RIG-I, and results in mitochondria expressing accumulated IPS-1- around the RIG-I foci. IPS-1 may be redistributed, resulting in a local accumulation of IPS-1 on a mitochondrion (left). IPS-I may further segregate due to mitochondrial reorganization by fusion and fission (right). Local accumulation of IPS-1 may further recruit adaptors and protein kinases to activate antiviral signaling.

We demonstrated that the redistribution of IPS-1 is induced by various viral infections and 5′ppp-RNA transfection and is dependent on a functional MFN1. The precise mechanism of the IPS-1 redistribution is not known, however we propose a model described in Fig. 13. In uninfected cells, MFN1 and IPS-1 associate constitutively (Fig. 10) and distribute evenly on all mitochondria. When a virus replicates in a specific cytoplasmic compartment, RIG-I is recruited to this area as a result of interaction with viral dsRNA through the C-terminal RNA-binding domain (Fig. 5A). Upon binding with viral RNA, CARD is exposed (activate RIG-I) [27], and then interacts with IPS-1 at the periphery of the viral compartment. At the same time, mitochondria surround the viral compartment because it lacks the ability to penetrate it (Fig. 6). Since mitochondrion is an elastic, movable organelle [28], affinity between RIG-I and IPS-1 may be sufficient for the mitochondrial relocalization. IPS-1/MFN1 complex may facilitate fusion between the surrounding mitochondria. Mitochondrial fission occurs independently to balance the fusion, however since the fusion of IPS-1/MFN1-enriched mitochondria facilitates the redistribution of IPS-1, IPS-1-enriched mitochondria may be generated. Further research is necessary to elucidate if the mitochondrial fusion process is indeed central to IPS-1 redistribution.

In summary, our study provides new insight into why the mitochondrial localization of IPS-1 is essential to its function. We demonstrated that MFN1 regulates the redistribution of IPS-1 in a RIG-I-signal-dependent manner. The mitochondrion provides a platform for the coordination of antiviral signaling by receiving the initial signal from the activated RIG-I. The signal is amplified through the accumulation of IPS-1, and other essential molecules such as tumor necrosis factor receptor-associated factors (TRAFs) and signaling protein kinases are recruited to mobilize active transcriptional regulators. In this regard, another organelle, the late endosome, functions similarly as a platform for antiviral signaling by recruiting different signaling components. This is initiated by Toll-like receptors (TLR3, 7, 8, and 9), another subtype of nucleic acid sensors.

## Materials and Methods

### Cell culture and transfection

HeLa, SKHep1, MEF, and 293T cells were maintained in Dulbecco's modified Eagle's medium with 10% fetal bovine serum and penicillin–streptomycin (100U/ml and 100µg/ml, respectively). L929 cells were maintained in minimum essential medium with 5% fetal bovine serum and penicillin-streptomycin. Immortalized wild-type MEFs and MEFs deficient in Mfn1 or Mfn2 were obtained from Prof. David Chan (Caltech). HeLa, L929, and 293T cells were transfected with Lipofectamine 2000 (Invitrogen). Stable transformants of IPS-1-HeLa cells were established by transfection of a linearized empty plasmid (pEF-Tak) or expression plasmid for FLAG-IPS-1 (pEF-Tak-FLAG-IPS-1), and selected with G418 (1mg/ml). We used HeLa IPS-1#2 clone for most experiments because IPS-1#2 clone showed higher induction of IFN among the stable clones, but we confirmed that other clones also showed the same phenotype. IPS-1/RIG-Iwt and IPS-1/RIG-I K270A-expressing HeLa cells were generated by transduction using a lentivirus system. cDNA for RIG-I wt or RIG-I K270A was cloned into the multi-cloning site of a lentiviral vector, pCSII-CMV-MCS-IRES2-Bsd. The recombinant lentiviruses were generated by co-transfection of the lentiviral vector together with lentivirus constructs, pCMV-VSV-G-RSV-Rev and pCAG-HIVgp, into 293T cells. At 48 h post-transfection, the culture supernatant was collected, and then added to IPS-1#2 HeLa cells. Three days later, the cells were selected with Blasticidin (10µg/ml).

### Viral infection and plaque assay

Cells were treated with culture medium or infected with SeV, NDV, Sindbis virus, EMCV, Influenza virus, or VSV at a MOI of 1 or 0.5 for qRT-PCR or immunofluorescence, respectively. The yield of EMCV in the culture supernatant was determined with a standard plaque assay [17].

### Plasmid constructs

pEF-Bos-FLAG-RIG-I CARD and pEF-Bos-FLAG-IPS-1 have been described previously [6], [17]. pEF-Tak and pEF-Tak-FLAG-IPS-1 were kindly provided by Dr. M. Gale (University of Washington School of Medicine, USA). pEF-Bos-HA-MFN1 and pEF-Bos-HA-MFN2 were newly constructed. MFN1 and MFN2 cDNA were amplified with a pair of oligonucleotides designed to add an N-terminal HA tag by PCR, and the PCR fragment was inserted into pEF-Bos(+). MFN2 cDNA was purchased from the Biological Resource Center of the National Institute of Technology and Evaluation of Japan. pEF-Bos-HA-MFN1 T109A was constructed with a KOD-Plus-Mutagenesis kit (TOYOBO, Japan). The nucleotide sequences for the constructs were confirmed with the BigDye DNA sequencing kit (Applied Biosystems). Luciferase reporter containing human IFN-β promoter/enhancer (−125-Luc) is described elsewhere [29].

### Synthetic RNA

Nucleotide sequences of the synthetic RNA were described previously [14]. 5′ppp-RNA was synthesized by in vitro transcription using the T7 Megascript kit (Ambion). 5′OH-RNA was chemically synthesized (Japan Bio Services Co., Ltd, Japan) and synthetic RNA was transfected by Lipofectamine RNAiMax (20 pmol in a 24-well format).

### Immunoblotting, luciferase assay, and antibodies

The preparation of cell extracts, luciferase assay, and immunoblotting have already been described [17], [29]. The antibodies used are: anti-FLAG antibody (SIGMA: F3165 and Affinity Bioreagents: PA1-984B), anti-ERAB antibody (Abcam: ab10260), anti-MFN1 antibody (Santa cruiz: sc-50330), anti-ACTIN antibody (Millipore: MAB150 1R), anti-MFN2 antibody (abcam: ab56889), anti-OPA1 antibody (abcam: ab42364), anti-DRP1 antibody (abcam: ab56788), and anti-BCLXL (Santa cruiz: sc-8392). The anti-NP antibody was produced by Dr. Y. Nagai, and provided by Dr. T. Sakaguchi (Hiroshima University, Japan). The anti-RIG-I antibody was generated by immunizing a rabbit with a synthetic peptide corresponding to a.a. 793–807 of RIG-I. The generation of the anti-IPS-1 guinea pig antibody has already been described [30]. Briefly, anti-IPS-1 rabbit antibody was generated by immunizing a rabbit with a recombinant protein corresponding to a.a. 1–157 of IPS-1.

### RNAi

The siRNA negative control and siRNAs targeting MFN1, OPA1, or DRP1 were purchased from Invitrogen, and transfected with RNAi MAX (Invitrogen) according to the manufacturer's recommendation (final concentration of siRNA was 50nM). At 48 or 72 h post-transfection, cells were harvested or infected with NDV or SeV, then subjected to qRT-PCR, immunofluorescence, or SDS-PAGE followed by immunoblotting.

### Quantitative real time PCR and microarray analysis

Total RNA was prepared with TRIZOL (Invitrogen) and treated with DNase I (Roche Diagnostics). A High-Capacity cDNA Reverse Transcription Kit (Applied Biosystems) was used for cDNA synthesis, and mRNA level was monitored with the Step One plus Real Time PCR system and TaqMan Fast Universal PCR Master Mix (Applied Biosystems). TaqMan primer-probes for human *IFNB1*, human *OPA1*, murine *Ifna4*, murine *Ifnb1*, and 18s rRNA were purchased from Applied Biosystems. The RNA copy numbers were normalized to that of internal 18s rRNA. In the microarray analysis, we used the Genopal microarray system according to the manufacturer's instructions (Mitsubishi Rayon). Biotin-labeled RNA was prepared with a MessageAmp II-Biotin Enhanced kit (Ambion).

### Immunofluorescence and immunoelectron microscopy

For immunofluorescence analysis, cells were fixed with 4% paraformaldehyde for 10 min, permeabilized with an acetone: methanol (1∶1) solution, and blocked with 5mg/ml of BSA in PBST (PBS, 0.04% Tween20) for 1 hour. The cells were next incubated with relevant primary antibodies overnight at 4°C, and then incubated with relevant Alexa Fluor 405, Alexa Fluor 488, or Alexa Fluor 594-conjugated secondary antibodies. Mitochondria were stained with MitoTracker Red CMXRos according to the manufacturer's instructions (Molecular Probes). Nuclei were stained with DAPI (4.6-diamidino-2-phenylinodole). The fluorescence image was quantified by software provided by Leica Microsystems. The percentage of IPS-1 redistribution among NDV-infected cells was scored by 3 persons, and data are shown as means ± s.d. of the three independent scores. Cells were analyzed with a Leica confocal laser-scanning microscope (TCS-SP2). For immunoelectron microscopy, cells were fixed with 4% paraformaldehyde and 0.05% and 0.01% glutaraldehyde for 10 min to detect NP and the FLAG tag, respectively, and then incubated with PBS containing 20% normal goat serum (NGS) and 0.075% Photo-Flo (Kodak) for 30 min at room temperature. The cells were incubated with the anti-NDV NP antibody or anti-FLAG antibody in PBS containing 2% NGS and 0.075% Photo-Flo overnight. After several washes with PBS, the cells were further incubated with a 1.4 nm gold-conjugated anti-mouse IgG goat IgG Fab fragment (Nanoprobes) in PBS containing 2% NGS and 0.075% Photo-Flo overnight. They were then washed with PBS and postfixed with 0.1 M phosphate buffer containing 1% (v/v) glutaraldehyde for 10 min at room temperature. After washing in distilled water, the gold particles were silver-intensified with an HQ silver kit (Nanoprobes) for 10–15 min. Then, the immunostained cells were incubated with 0.5% osmium tetroxide in 0.1M phosphate buffer for 40 min at room temperature. After dehydration with ethanol, cells were embedded in epoxy resin (Luveak 812; Nacalai Tesque, Japan). Once the resin was polymerized, the cells were cut into ultrathin sections on an ultramicrotome, Reichert-Nissei Ultracut S (Leica). The ultrathin sections were mounted on mesh grids, and stained by the Reynolds method. Finally, the ultrathin sections were examined with an electron microscope (H-7650; Hitachi).
